# The relationship between manuscript title structure and success: editorial decisions and citation performance for an ecological journal

**DOI:** 10.1002/ece3.1480

**Published:** 2015-04-16

**Authors:** Charles W Fox, C Sean Burns

**Affiliations:** 1 Department of Entomology, University of KentuckyLexington, Kentucky; 2 School of Library and Information Science, University of KentuckyLexington, Kentucky

**Keywords:** Citation analysis, editorial review, *Functional Ecology*, peer review, writing style

## Abstract

A poorly chosen article title may make a paper difficult to discover or discourage readership when discovered, reducing an article's impact. Yet, it is unclear how the structure of a manuscript's title influences readership and impact. We used manuscript tracking data for all manuscripts submitted to the journal *Functional Ecology* from 2004 to 2013 and citation data for papers published in this journal from 1987 to 2011 to examine how title features changed and whether a manuscript's title structure was predictive of success during the manuscript review process and/or impact (citation) after publication. Titles of manuscripts submitted to *Functional Ecology* became marginally longer (after controlling for other variables), broader in focus (less frequent inclusion of genus and species names), and included more humor and subtitles over the period of the study. Papers with subtitles were less likely to be rejected by editors both pre- and post-peer review, although both effects were small and the presence of subtitles in published papers was not predictive of citations. Papers with specific names of study organisms in their titles fared poorly during editorial (but not peer) review and, if published, were less well cited than papers whose titles did not include specific names. Papers with intermediate length titles were more successful during editorial review, although the effect was small and title word count was not predictive of citations. No features of titles were predictive of reviewer willingness to review papers or the length of time a paper was in peer review. We conclude that titles have changed in structure over time, but features of title structure have only small or no relationship with success during editorial review and post-publication impact. The title feature that was most predictive of manuscript success: papers whose titles emphasize broader conceptual or comparative issues fare better both pre- and post-publication than do papers with organism-specific titles.

## Introduction

Nearly all published scientific papers have a title, and this title is the first part of the paper that prospective readers encounter. The title gives readers a summary of the content of the manuscript, provides keywords and index terms in electronic databases (making an article findable; Rodríguez and Moreiro 1996; Beel and Gipp 2009), and motivates (or not) prospective readers to read an article (Diener 1984; Ball 2009). A poorly chosen title can make a paper hard to discover or ignored when discovered, either of which will substantially reduce its impact on the scientific community. A manuscript's title is also the first point of contact between a paper and prospective peer reviewers. Reviewers are generally sent the manuscript title when invited to review a paper; their first impression of the paper, and whether or not they agree to review, may be influenced by features of the title.

Titles of scientific papers have been changing in structure over time. For example, the use of subtitles and the word count of titles have both been increasing in many journals (Lewison and Hartley 2005). Yet whether and how the structure of a manuscript's title affect readership and impact of a paper remain unclear. Advances in information retrieval, with search engines generally including keywords, abstracts, and often the full text of papers, have likely reduced the need for informative titles to maximize information retrieval (Rostami et al. 2014). However, titles still need to attract reader attention and stand out in database search results and electronic tables of contents. Likely as a consequence, we have seen an increase in the usage of questions and of wit in scientific titles – marketing strategies to attract readers rather than inform them of a paper's content (Ball 2009). However, titles containing wit, acronyms, exclamations, questions, and metaphors often inaccurately describe a paper's content (Aleixandre-Benavent et al. 2014) and may signal frivolity and lack of credibility (Francl 2014). Thus, such papers may have lower impact and be cited less, despite being downloaded more (Sagi and Yechiam 2008).

In this study, we examine (1) how the features of manuscript titles (word count, title features, and the usage of humor) have changed over time, and (2) whether the structure of a manuscript's title is predictive of success of a manuscript during the manuscript review process and impact of a manuscript (using citations counts as a proxy) after publication. Our study is unusual in that we make use of a comprehensive dataset including detailed peer-review data for all manuscripts submitted to one specific ecological journal, *Functional Ecology*, from 2004 to 2013 (inclusive). We then use publicly available citation data (*Web of Science*) to quantify the relationship between title structure and citations for papers that are published by this same journal, *Functional Ecology*, from 1987 (volume 1) to 2011 (volume 25).

## Methods

### Peer-review dataset

**Figure 1:**
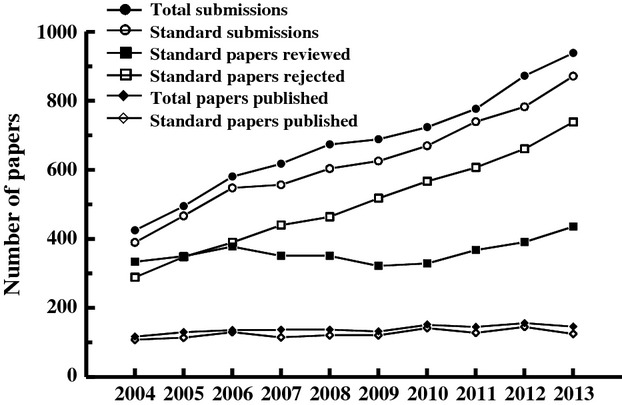
The number of papers submitted to, reviewed by, rejected from, and published in *Functional Ecology* between 2004 and 2013. “Total submissions” and “Total papers published” include all papers (invited papers, reviews, perspectives, and other paper types), whereas “Standard submissions” and “Standard papers published” include just research papers (those used for most of the statistical analyses presented in this paper, as described in the Methods). Papers reviewed and rejected is a subset of standard papers, not total papers. Papers published in a particular year are primarily papers submitted the previous year and thus overlap but are not the same as papers accepted in that particular year.

*Functional Ecology* uses ScholarOne Manuscripts (previously Manuscript Central) to manage manuscript submissions and peer review. We extracted data from ScholarOne (on 19 December 2014) for all “standard” papers submitted to *Functional Ecology* between 2004 and 2013 (inclusive). “Standard” papers include all typical research studies (empirical or theoretical), but exclude review papers, commentaries, perspectives, editorials, and other types of papers not considered typical research manuscripts. Over this time period (2004–2013), the journal received 6795 total submissions, of which 6257 were standard papers included in our analyses (Fig.1). Of these standard papers, 3610 were sent out for peer review.

The editorial review process is generally broken into two steps at *Functional Ecology*. All papers are first evaluated by an editor in a pre-peer review assessment of the suitability of a paper for *Functional Ecology*. A subset of all submissions are declined at this stage. Remaining papers are sent for peer review, followed by another editorial assessment and rendering of a final decision. Decisions can include reject, request for revision, or accept (although “accept” is almost never used for standard papers until after a round of revision). For our analyses, we examined these two steps separately, with all papers being categorized as “reviewed” or “not reviewed” after the first stage, and then “rejected” or “not rejected” after the second stage.

We only examine the fate of papers during their initial submission to the journal; a small subset of papers, only 1–2% of invited revisions, are rejected after revision, but these papers are treated as “not rejected” in our analysis because revision was invited. Papers that are rejected but with resubmission invited (commonly called “reject without prejudice”) are counted twice in our dataset (if resubmitted) because each submission has a unique manuscript number and is treated editorially as a separate paper. Second submissions have a much higher success rate than normal submissions (because they were invited) and thus create a potential bias in our dataset. We considered deleting these papers from the dataset, but this was problematic because paper titles and (less often) author lists can change between the first and second submission, making identifying resubmitted papers difficult. However, only 2.7% of submissions in our dataset were “reject with resubmission” decisions, such that any potential error is at most small. Our analysis does account for papers whose initial decision was overturned on appeal (i.e., reject changed to revision) because this is updated in ScholarOne; our analysis thus uses the final decision postappeal rather than the pre-appeal decision, except for rare cases where such papers are resubmitted as new manuscripts and thus have a new manuscript number.

Our dataset also includes how many reviewers were invited for each paper, how many agreed/declined to review (or failed to respond), and how long the peer-review process took for each individual paper.

### Citation dataset

Citation data were extracted via the Web interface available to library subscribers of *Web Of Science* on 17 December 2014. For our analyses of how title structure has changed over time, we include data on all published standard papers (defined above) from 1987 (volume 1) to 2014 (volume 28) (inclusive). However, for analyses of citation counts we only include standard papers published between 1987 and 2011. We exclude papers published after 2011 (2012 to present) because citation counts were generally low with a lot of papers yet to be cited.

*Functional Ecology* published a total of 2785 papers during the 25-year period included in the citation analysis, of which 2435 were standard papers and included in our analyses of citation counts. An additional 458 papers were published in 2012–2014, of which 415 were standard papers and included in our analysis of the change in title structure over time, but not the analysis of citations.

### Categorizing titles

Our focus in this study is on the structure of titles. Titles from the *Web of Science* database are as published by the journal, but all titles in the ScholarOne database have been entered by authors. A small subset of author-entered titles are known to deviate slightly from the actual title of the submitted paper as printed on the document (e.g., MS Word or pdf file) submitted by the author. Unfortunately, we cannot access all of the original manuscripts to reconcile such discrepancies – older documents are archived by ScholarOne and only some are available without a substantial per-manuscript fee. We did, however, proof the entire dataset for typos in titles that would affect word count or other classification variables described below.

Titles were categorized by hand as being compound titles (having a subtitle, yes/no) or being a question versus statement. We also categorized titles as being amusing or not. Amusing titles generally had subtitles; in most cases, the amusing part of the title was before the colon, with the subtitle presenting a more typical serious title. Titles were categorized as amusing if one part of the title (before or after the colon) conveyed little or no information about the content of the paper but instead appeared (to CWF and CSB) to be a metaphor, a pun or double entendre, or a play on famous quotes, titles, or other phrases in common parlance.

One question of interest is whether papers written to be understandable by a narrow versus broad readership fare better during peer review and have more impact post-publication. A proxy for this is whether a title includes reference to a specific study organism by its taxonomic name rather than reference to more widely recognizable common names or higher level taxonomic categories. We thus scored whether titles had a specific organism indicated in the title by genus or species name (e.g., *Callosobruchus* or *Callosobruchus maculatus*) versus more general titles that did not reference a particular genus or species by their taxonomic name; the latter category includes papers with no organismal reference and those that reference organisms by higher taxonomic groupings (e.g., families, orders) or use common names. One special case is the group Drosophila (the most common group of organisms referenced in titles), which is both the taxonomic name (genus) and the common name of a group of fruit flies; because we cannot identify author intent from their titles, we treated *Drosophila* as a genus and thus categorized titles including this name as referencing a particular genus or species.

### Statistical analyses

All statistical analyses presented here include only standard research papers as defined above, that is, we exclude reviews, perspectives, editorials, or other types of nonresearch papers.

We used logistic regression to analyze how title structures changed over time (frequency of questions, subtitles, or amusing titles), for example, Subtitles - SubmissionYear. We also examined the fate of submitted manuscripts using logistic regression, but in two sequential analyses, first examining whether a paper is rejected *before* peer review (rejected - 1, reviewed - 0) and then, if a paper is sent for peer review, whether it was rejected or not (rejected - 1, not rejected - 0). Both models were of the form Reject - SubmissionYear + Question + Subtitle + SpeciesNames + TitleWordCount, with SubmissionYear, Question, Subtitle, and SpeciesNames as categorical variables (0 vs. 1), and TitleWordCount as a covariate (SAS PROC LOGISTIC). Because amusing titles generally contained subtitles, and subtitles were predictive of manuscript fate, we compared manuscript decisions for amusing versus nonamusing titles for the subset of titles that had subtitles, Reject - SubmissionYear + TitleWordCount + Amusing.

How the word count of paper titles changed over time, and whether the number of reviewers invited (ScholarOne dataset) and citation counts were influenced by title structure, were all analyzed using standard general linear models (GLM). Word counts met the assumption of GLM and were not transformed. However, citation counts deviated substantially from the assumptions of GLM, with most papers having few citations and few papers having many citations (for skewness in citation distributions, see Seglen 1992). Citation counts were log-transformed, as log(citations+1), to meet as best as possible the assumptions of GLM.

Whether citation counts are affected by title structure was examined by (1) first identifying the terms included in the best fit model (SAS PROC GLMSELECT using AICc as our selection criterion), with the starting model Citations - PublicationYear + PageCount + TitleWordCount + Question + Subtitle + SpeciesNames, with PublicationYear, Question, Subtitle, and SpeciesNames as categorical variables, and + PageCount and TitleWordCount as covariates, then (2) individually adding terms not included in the best fit model (SAS PROC MIXED) to confirm they were nonsignificant and that the significance of terms in the best fit model did not change. Additional GLMs presented in the Results section were one-, two- or three-factor GLM models as described in the Results where presented.

## Results

### Journal submissions

Submissions of papers for consideration for publication in *Functional Ecology* increased substantially from 2004 (425 submissions) to 2013 (937 submissions), a total increase of 120% and an average increase of 9.2% per year over the time period included in the peer-review aspect of this study (Fig.1). This increase continued in 2014 (not included in this study), when the journal received 1048 submissions. Over this same period, the number of pages published by *Functional Ecology* increased from 959 pages in 2004 to 1454 pages in 2013, a 52% increase. This increase in pages allowed more papers to be accepted, although the increase in papers published was more modest, from 114 in 2004 (excluding errata and editorial material, but including forum and review papers) to 138 in 2013. Because submissions have been increasing much faster than the journal page allocations, editors have necessarily accepted a smaller proportion of submissions; in 2004, editors accepted 24% of submissions (excluding invited papers), whereas they accepted just 15% of submissions in 2013. Most of this difference in acceptance rate is due to stricter pre-review screening; editors sent 87% of submissions out for peer review in 2004, but sent only 50% out for review in 2013. Of papers sent for peer review, 27% were accepted in 2004, whereas 30% of papers sent for review were accepted in 2013 (this value fluctuated between 27% and 38% [2006] over the 10-year period).

### Change in title structure over time

#### Submitted papers

**Figure 2:**
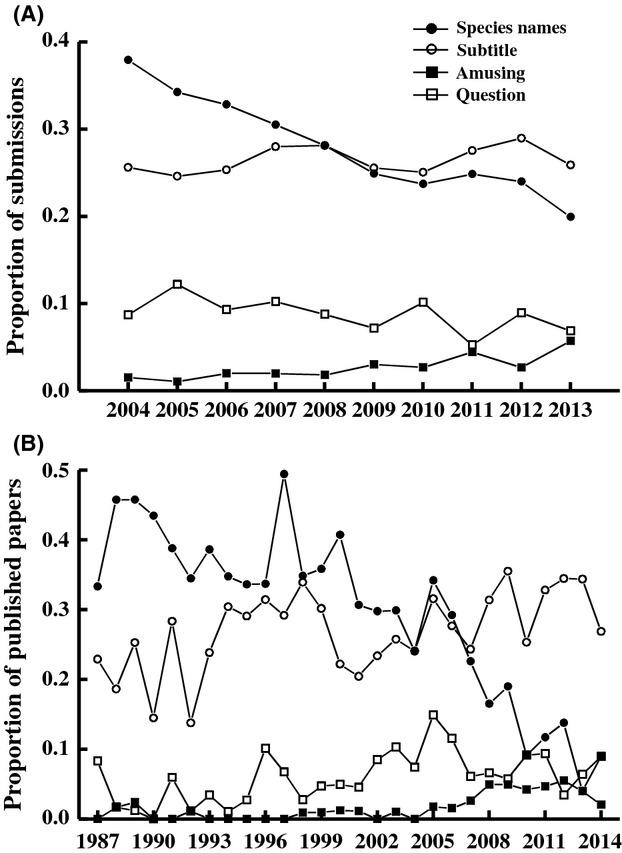
The proportion of standard papers that include specific features in their titles for papers (A) submitted to and (B) published in *Functional Ecology*. Values are the proportion of all standard papers submitted.

Titles of papers submitted to *Functional Ecology* changed over the course of the study (2004–2013) (Fig.2). The proportion of submissions that included a genus or species name in the title declined, as did the proportion of submissions that had a question in the title, over the 10 years included in this study (logistic regression, model: TitleFeature - Year, with year as a covariate; 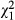 - 75.2, *P* < 0.001 and 9.4 and *P* - 0.002 for species names and questions, respectively). In contrast, the proportion of submissions that had amusing titles increased over time (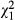 - 29.1, *P* < 0.001), although this effect was largely in just the last few years (Fig.2A). There was no significant change in the proportion of submissions that included a subtitle (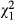 - 0.78, *P* - 0.38). There was also no statistically significant change in average word count of titles over the period of the study (*F*_1,6255_ - 2.38, *P* - 0.12). However, titles were on average longer for papers that included a genus or species name (comparing least-squares means, 2.1 words longer; *F*_1,6253_ - 343.8, *P* < 0.001) or had subtitles (2.3 words longer, *F*_1,6253_ - 422.7, *P* < 0.001). After removing these effects, we observed that title word counts increased slightly but significantly over time, a total change of ∼0.6 words (based on least-squares means) over the period of the study (model: TitleWordCount - Year + Subtitle + SpeciesName, Year effect: *F*_1,6253_ - 11.8, *P* < 0.001).

#### Published papers

The structure of manuscript titles has changed over the course of the study for the subset of papers actually published by *Functional Ecology* (all standard papers published 1987–2014) (Fig.2B). The frequency of subtitles and questions in titles both increased (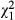 - 6.55 and 21.4, respectively, *P* < 0.02 for each), as did the frequency of amusing titles (of papers including a subtitle; 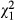 - 19.7, *P* < 0.001). In contrast, the frequency of genus and species names decreased over time (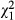 - 87.0; *P* < 0.001). As with journal submissions, title word counts were greater for papers with subtitles (by ∼ 2.4 words, comparing least-squares means) and for papers including genus or species names (by ∼2.2 words) than for papers without (*F*_1,2430_ - 187.7 and 158.6, respectively, *P* < 0.001 for each). Titles containing questions did not differ in length from titles written as statements (*F*_1,2430_ - 0.47, *P* - 0.49). The overall word count of titles did not change across time (*F*_1,2433_ - 2.44, *P* - 0.12) but, when controlling for the change in frequency of subtitles and genus/species names in the analysis, we see that word counts of titles increased slightly but significantly, by approximately one word over 25 years (*F*_1,2431_ - 11.2, *P* < 0.001).

### Title structure and the peer-review process

**Figure 3:**
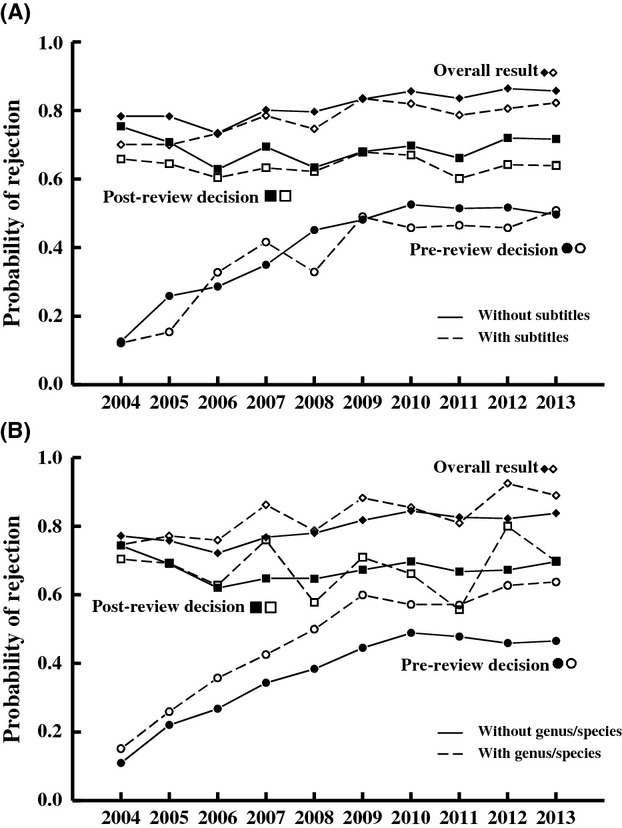
The relationship between title features and the fate of manuscripts submitted to *Functional Ecology* that contain (A) a subtitle or (B) the specific name of a focal research organism (genus or species names). The probability of reject at the “post-review decision” stage is the probability of rejection for the subset of papers that were sent for review (i.e., the papers not rejected at the pre-review decision stage). The “overall result” is the cumulative probability of reject at any stage.

**Figure 4:**
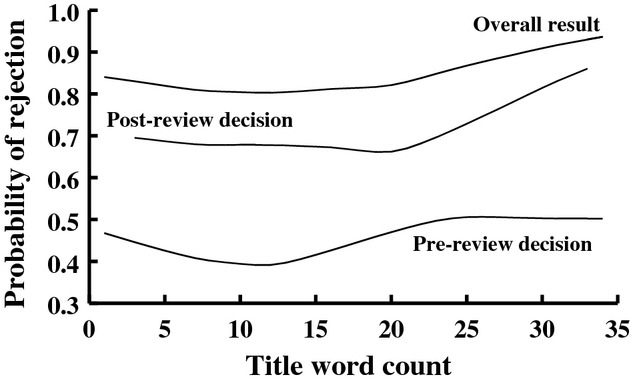
Cubic spline fit (with binomial data) for the probability of rejection versus title word count for papers submitted to *Functional Ecology* between 2004 and 2013.

Three features of titles were predictive of the fate of a paper throughout the editorial process. Papers with subtitles were more likely to be sent for review than papers without subtitles (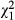 - 5.54, *P* - 0.02) and were more likely to be accepted if sent for review (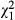 - 10.2, *P* - 0.001) (Fig.3A). Papers containing genus and species names were substantially *less* likely to be sent for peer review than were papers without genus or species names (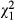 - 43.8, *P* < 0.001), although there was no difference in rejection rate between these two types of papers if they were sent for peer review (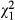 - 0.01, *P* - 0.95) (Fig.3B). This negative effect of taxonomic information in the title was also seen for titles using less specific taxonomic references (common names and/or higher order taxonomic categories); papers with such titles were less likely to be sent for peer review (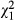 - 5.41, *P* - 0.02) and more likely to be rejected if sent for review (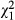 - 5.45, *P* - 0.02) compared to papers with no organismal reference in their title. Finally, title word count was predictive of a paper's fate. Papers with overly long titles fared especially poorly, although the effect was not linear; papers with intermediate length fared the best through the peer-review process (Fig.4). We found no evidence that titles written as questions or that were amusing fared differently than papers with nonquestion or nonamusing titles (pre-review screening: 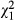 - 0.57, *P* - 0.45 and 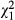 - 2.14, *P* - 0.14 for questions and amusing titles, respectively; post-review decision: 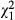 - 1.54, *P* - 0.21 and 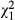 - 1.91, *P* - 0.17).

**Figure 5:**
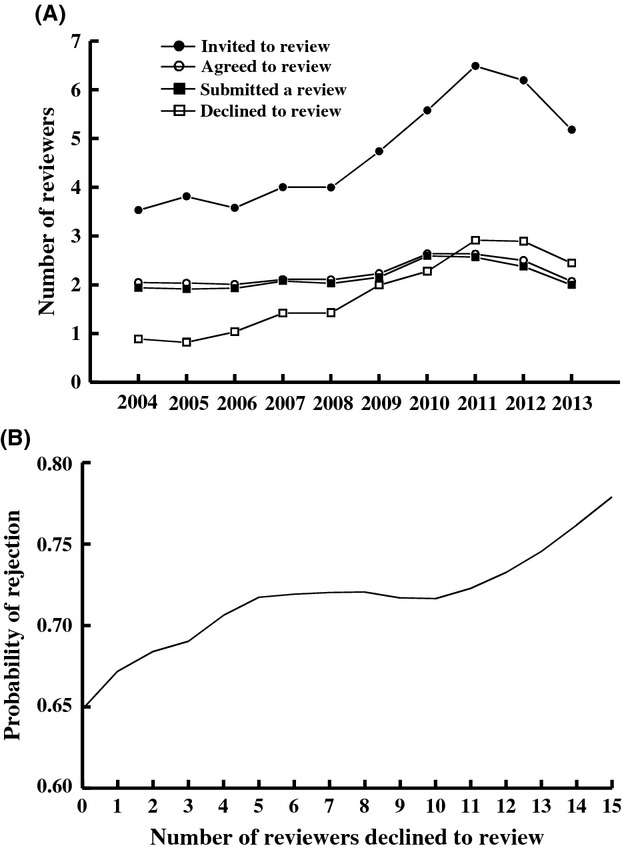
(A) The number of reviewers invited, agreed to review, submitting a review, and declining to review from 2004 to 2013. Note that *Functional Ecology* editors have had to invite increasingly more reviewers to obtain approximately the same number of reviews (two per paper) over the course of the 10-year period. (B) Cubic spline fit (with binomial data) for the probability of rejection versus the number of reviewers that declined to review a manuscript. The analysis controls for the effect Submission Year.

Finding reviewers for *Functional Ecology* manuscripts has become substantially more difficult for editors over the 10 years of the study. In 2004, editors invited an average of 3.53 reviewers per paper, of which 2.05 agreed to review, and 1.94 submitted their reviews. In 2013, editors needed to invite on average 5.18 reviewers (47% more than in 2004) to have on average 2.07 agree to review and obtain 2.00 reviews per paper. This difference in the number of reviewers invited is driven entirely by an increase between 2004 and 2013 in the proportion of invited reviewers that decline to review; in 2004, just 0.89 invited reviewers on average declined per paper whereas 2.45 declined per paper in 2014 (Fig.5A) (reviewers that do not respond to queries, or that do not respond in a timely fashion, are considered as “no response” and not as “declines”). We found no evidence that the structure of the title is correlated with how many reviewers the editors have to invite to get two reviews, how long it takes reviewers to respond to our query or how long it takes to reach a decision on a reviewed paper (“time to decision” includes both reviewer and editor handling time). However, the number of reviewers that editors have to invite for a paper is predictive of its fate – the probability that a paper was rejected after review *increased* with the number of reviewers that declined to review it, even after controlling for the effect of submission year (Fig.5B) (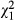 - 8.77, *P* - 0.003).

### Title structure and post-publication impact of papers

**Figure 6:**
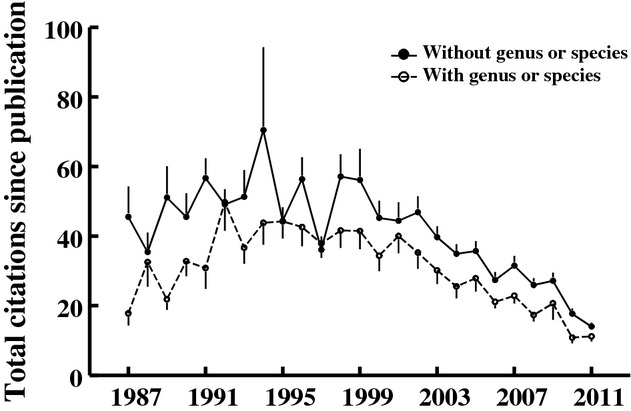
The relationship between a reference to a specific study organism by taxonomic name (genus or species name) and total citations (±SEM) received for papers published in *Functional Ecology* between 1987 and 2011.

The best fit statistical model describing the number of citations a paper received (total citations received for standard papers published 1987–2011 inclusive) included only the year of publication (older papers received more citations; *F*_24,2408_ - 22.9, *P* < 0.001), paper length (longer papers received more citations; *F*_1,2408_ - 133.1, *P* < 0.001) and the presence/absence of a species name in the manuscript title (Fig.6; papers including genus or species names were cited on average 6.5 fewer times than papers with less specific titles; *F*_1,2408_ - 44.1, *P* < 0.001). Title word count and whether the paper title is a question or contains a subtitle did not influence the number of citations a paper received; these terms were not present in the best fit model and were nonsignificant when added individually to the best fit model (*F*_1,2407_ - 0.83, *F*_1,2407_ - 0.19, and *F*_1,2407_ - 2.21, *P* > 0.13 for each). Of papers with subtitles, there was no evidence that papers with amusing titles were cited differently from papers without amusing titles (*F*_1,653_ - 2.61, *P* - 0.11).

## Discussion

We found that titles of manuscripts submitted to and/or published by *Functional Ecology* have changed in structure over time – titles became marginally longer (after controlling for other variables), the usage of humor increased, and the inclusion of specific names (genus and species names of study organisms) in titles decreased over the period of the study. The frequency of subtitles in submissions did not change between 2004 and 2013 (the period covered by our peer-review dataset), but subtitles did increase in frequency among published papers throughout the longer history of the journal (starting in 1987). Papers with subtitles fared better than papers without through editorial and peer review, whereas papers with specific taxonomic names fared poorly during editorial (but not peer) review. Papers with very short titles, and especially those with very long titles, fared more poorly during editorial review than did papers with intermediate length titles. We found no evidence that the structure of titles affects the ability of editors to recruit reviewers for peer review. Only one feature of titles – the presence of specific names – was predictive of manuscript impact; papers containing specific names were less well cited, on average, than were papers lacking specific names.

The most striking result of our analysis is that papers containing a genus or species name in their title – those referencing a specific study organism by its taxonomic name – fared more poorly during editorial review (Fig.3B) and were less frequently cited after publication (Fig.6), compared to papers lacking such specific taxonomic names. Also, the frequency of specific names in titles has declined substantially among submitted papers over the 10 years between 2004 and 2013. Papers with titles written to emphasize the broader context of their study likely appeal to a broader segment of a journal's readership and are thus more widely read and cited. Some data support this; for example, biomedical papers referencing a specific geographic region or study population in their titles are less widely cited than are papers lacking reference to a specific locality (Jacques and Sebire 2010; Paiva et al. 2012). We interpret our results as analogous to this, though for ecological (nonhuman) rather than biomedical (human) studies. However, we cannot distinguish whether this is a causal relationship, for example, specific names in paper titles signal to editors and readers that the paper is not of general interest, or whether title structure simply covaries with other features of the paper, for example, actual narrow context for the study, that lead to poor outcomes. The lack of a relationship between this title feature and the willingness of reviewers to review a paper suggests the latter – that there is no cause-effect relationship between the specificity of the title and reviewer interest in a paper, but this is confounded by the specialized interest of scientists invited to review for the journal (see below). We need readership data (e.g., downloads) rather than citation data to tease apart paper title and paper content effects on citations. That the frequency of specific names in titles has declined dramatically for both submitted and published papers at *Functional Ecology* almost certainly reflects the growing competitiveness of high impact factor ecology journals. Submissions have been increasing more quickly than page allocations for many of the top impact journals and thus the criteria for acceptance have gotten more stringent. As a consequence, papers that do a poor job of placing work in a broad conceptual or comparative context probably fare poorly at most top impact journals, and certainly do at *Functional Ecology*, because these journals preferentially publish papers expected to have broad impact on the field. It is thus likely that the decline in frequency of specific names in titles reflects a growing understanding by authors that papers, or at least titles, need to emphasize broader issues to appeal to editors and likely also to attract readers.

Guides to writing scientific papers generally suggest that concise (but informative) titles are preferable to longer titles (e.g., Gasparyan et al. 2011; Mack 2012; Grant 2013; Liumbruno et al. 2013; but see Kumar 2013). Longer titles, though, can be more informative, have more searchable key words, and so can be more easily discovered and thus potentially more widely read, especially in databases that place weight on title keywords for information retrieval (Beel and Gipp 2009). Some evidence indicates that titles have been increasing in length over time (Lewison and Hartley 2005; Webster et al. 2009; Whissell 2013), although this varies among journals (Méndez et al. 2014). We found that title word count of papers submitted to and published by *Functional Ecology* increased only very slightly over time, an effect only observable after controlling for changes in other features of titles. Increasing word counts of titles over time may reflect increasing complexity of scientific disciplines (White and Hernandez 1991) and the trend toward an increase in the number of authors on papers (White 1991; Yitzhaki 1994). But studies are mixed on whether papers with longer or shorter titles are cited more; some have found that title word count does not predict the number of citations a paper will receive after publication (Stremersch et al. 2007; Haslam et al. 2008; Jamali and Nikzad 2011; Falagas et al. 2013; Rostami et al. 2014), whereas a few have found that papers with shorter titles (Paiva et al. 2012) or longer titles (Habibzadeh and Yadollahie 2010; Jacques and Sebire 2010) are more highly cited. The specific relationships appear to vary quite substantially among journals (Habibzadeh and Yadollahie 2010) and disciplines (van Wesel et al. 2014) with effect sizes always being quite small. Unfortunately, few studies distinguish types of papers, for example, review papers versus standard research papers. We limited our analysis of *Functional Ecology* papers to standard research papers and found that title word count did not predict the number of citations received after publication, but it is predictive of success during editorial review. Papers with long titles fared most poorly (there was an overall positive relationship between title word count and likelihood of rejection of a paper), but papers with very short titles also fared more poorly during editorial review (Fig.4). We doubt that title word counts causally affect editorial rejections; instead, it is likely that overly short titles or overly long titles reflect either lack of familiarity of authors with the type of journal they are submitting their paper to, or lack of care in preparation of the manuscript, both of which lead to problems in the full paper that are reflected in the choice of title. Unfortunately, disentangling causal versus correlative relationships requires objective assessment of manuscript quality and significance that is assessed independently of the title, something well beyond the scope of our study.

Modern advances in information retrieval have likely reduced the importance of informative titles to maximize information retrieval, but titles still must stand out in database results lists and capture the interest of prospective readers. Titles thus need to be not only informative, but also interesting. Likely to make papers more interesting, the use of sensationalist language in titles has increased across the full scientific literature since the 1950s (Jasienski 2009). The use of humor (Whissell 2009) and questions (Ball 2009) in paper titles have also increased, and this trend may be exaggerated by the recent dissemination of article titles on social media that authors use to promote or market their research (Thelwall et al. 2013; Cronin 2014). However, there is little evidence that sensationalist titles increase citations (Jasienski 2009), and amusing titles have been found to have at most a weak positive or a strong negative relationship (Sagi and Yechiam 2008) to citations, depending on the level of amusement. Articles with questions in the title have been found to be downloaded more (Jamali and Nikzad 2011) or the same (Paiva et al. 2012), but are subsequently cited less (Jamali and Nikzad 2011; Paiva et al. 2012) than are papers with descriptive or declarative titles. We found that the proportion of papers submitted to *Functional Ecology* with questions in their titles varied among years from ∼5% to ∼12%, but we found no evidence that papers with questions in their titles had different success rates during editorial review or were cited differently after publication.

The use of subtitles in papers has increased between the early 1980s and early 2000s throughout most areas of scientific publishing (Lewison and Hartley 2005). Consistent with this, we saw an increase in the frequency of subtitles in papers published by *Functional Ecology* over the study period 1987–2011, although this increase was not observed in the recent 10 years of submissions to the journal (through 2013; Fig.2A). Papers with subtitles are generally longer (more words) and can be both appealing and informative because they generally contain both a general (often before the colon or dash) and more specific (after the colon) component (Hartley 2007). There is experimental evidence that academics prefer titles with colons (Hartley 2007). However, as with the other variables examined here, the relationship between the presence of subtitles and citations is mixed. Papers with subtitles (Jacques and Sebire 2010; Rostami et al. 2014), or with nonalphanumeric characters indicative of subtitles (colons and dashes; Buter and van Raan 2011; Haslam et al. 2008), have been found to be cited more often than are papers without subtitles, but other studies have also found either no relationship (Hartley 2007) or the opposite relationship (Jamali and Nikzad 2011; Paiva et al. 2012). We found that papers with subtitles were less likely to be declined by *Functional Ecology* editors before review and less likely to be declined if sent for review, but papers with subtitles did not receive more citations if published.

For *Functional Ecology,* we found that longer papers (papers with more pages) were more highly cited than were shorter papers. This is consistent with a variety of previous studies (Haslam et al. 2008; Ball 2009; Vieira & Gomes 2010; Falagas et al. 2013; van Wesel et al. 2014; but see Jamali and Nikzad 2011), including one analysis of ecological studies (Leimu and Koricheva 2005). Greater length of a paper could reflect greater scientific complexity of the study, more effort given to discuss conceptual significance of the work, or simply that the paper contains more information and thus may be citable for a greater diversity of points made by other scientists (Falagas et al. 2013). Alternatively, longer papers tend to have more authors, and papers with more authors tend to be more highly cited (Leimu and Koricheva 2005). Unfortunately, our study cannot distinguish these possibilities. We also could not examine how manuscript length influences the outcome of editorial review because manuscript length data are not available (author-supplied word counts are inconsistent in what they count). We know, however, that editors of *Functional Ecology* are less likely to consider a paper if it is overly long, will occasionally return papers for shortening before review, and will commonly require shortening of papers after review. This is because the journal's publishing contract specifies a number of pages published per year, not papers published per year, and so editors are strict about manuscript page lengths to increase the number of papers accepted per year.

One particularly novel element of our study is our examination of how manuscript titles influence the ability to recruit reviewers for peer review. Like many scientific journals (Tite and Schroter 2007; Lajtha and Baveye 2010; Graur 2014; Merrill and Cox 2014), *Functional Ecology* has experienced substantially increased difficulty in recruiting people to serve as peer reviewers over the past 10 years (Fig.5A). When invited to review for *Functional Ecology*, prospective reviewers are provided the manuscript title, names of authors and the manuscript abstract. The intent is to allow prospective reviewers to self-assess their suitability as a reviewer and to decide if they have adequate interest in the specific topic of a paper to allocate their time to reviewing it. We found no evidence that the willingness of scientists to review a specific paper is related to any features of the paper's title. Even the presence of specific names (genus or species), which was predictive of whether a paper was sent for peer review, did not influence the willingness of reviewers to agree to a review request. This is likely because reviewers are a narrow subset of all scientists specifically because they have expertise and interest in the specific topic and/or organisms examined in the paper.

We did, however, find that the number of reviewers that declined to review a paper (until two reviewers agreed to review) was predictive of the fate of the paper (Fig.5B); the more reviewers that needed to be invited, the less likely the paper was to be accepted. This matches the anecdotal experiences of a number of ecology editors (C. Fox, personal observation). We can imagine two types of hypotheses that might account for this relationship. First, it is possible that difficulty finding reviewers is predictive of a paper receiving poor reviews. Such papers might receive poor reviews because editors must eventually recruit less suitable or less qualified reviewers (because the most qualified/preferred reviewers declined) who subsequently review papers less positively. Or such papers might receive poor reviews because they are lower quality papers that reviewers declined to review because of accurate indicators that the paper would be of poor quality (e.g., author reputation or content [but not structure] of the title). A second hypothesis is that difficulty finding reviewers creates bias in the editorial decision itself, separate from potential effects on the peer-review stage of the process. Difficulty finding reviewers may frustrate editors, or signal to editors (consciously or unconsciously) a problem with the paper, increasing the likelihood the paper is rejected. Because we examined editorial decisions, our data do not allow us to disentangle the relative effect of reviewer versus editor assessment; editorial decisions are influenced by but not the same as peer reviewer recommendations.

## Conclusion

The use of subtitles, questions, word count, and other features of *Functional Ecology* paper titles have changed over time. Despite a growing body of literature suggesting that specific features of titles are more appealing to authors, or aid in information retrieval, there is little agreement among studies about whether features of titles affect readership or impact of paper. When studies do find relationships between title features and impact, observed effects are quite small. Our data agree with this generalization. One exception seems to be that titles written to focus on narrow aspects of a study – the specific geographic region or population studied (for medical studies) or the specific study species (for ecological studies) – are less impactful than papers with titles that emphasize the broader conceptual or comparative context of a study. However, it remains unclear whether this reduced impact reflects cause-and-effect – title structure affects paper impact – or, as we suspect, that titles simply reflect the content of the paper and thus narrowly versus broadly focused titles accurately predict whether a paper will interest a broader readership.
